# Development of a nomogram to predict 30-day mortality of patients with sepsis-associated encephalopathy: a retrospective cohort study

**DOI:** 10.1186/s40560-020-00459-y

**Published:** 2020-07-02

**Authors:** Yang Yang, Shengru Liang, Jie Geng, Qiuhe Wang, Pan Wang, Yuan Cao, Rong Li, Guodong Gao, Lihong Li

**Affiliations:** 1 Department of Neurosurgery, Tangdu Hospital, Air Force Medical University, Xi’an, 710038 China; 2 Department of Endocrinology, Xijing Hospital, Air Force Medical University, Xi’an, 710032 China; 3 Department of Surgery, Tangdu Hospital, Air Force Medical University, Xi’an, 710038 China; 4 Department of Liver Disease and Digestive Interventional Radiology, National Clinical Research Centre for Digestive Disease and Xijing Hospital of Digestive Diseases, Air Force Medical University, Xi’an, 710032 China; 5 Institute of Medical Research Northwestern Polytechnical University, Xi’an, 710072 China; 6 Department of Neurosurgery, Xijing Hospital, Air Force Medical University, Xi’an, 710032 China; 7 Department of Neurosurgery, The 986th Hospital of Chinese People’s Libertation Army, Xi’an, 710054 China; 8 Department of Emergency, Tangdu Hospital, Air Force Medical University, Xi’an, 710038 China

**Keywords:** Sepsis, Sepsis-associated encephalopathy, 30-day mortality, Nomogram, RDW

## Abstract

**Background:**

Sepsis-associated encephalopathy (SAE) is related to increased short-term mortality in patients with sepsis. We aim to establish a user-friendly nomogram for individual prediction of 30-day risk of mortality in patients with SAE.

**Methods:**

Data were retrospectively retrieved from the Medical Information Mart for Intensive Care (MIMIC III) open source clinical database. SAE was defined by Glasgow Coma Score (GCS) < 15 or delirium at the presence of sepsis. Prediction model with a nomogram was constructed in the training set by logistic regression analysis and then undergone internal validation and sensitivity analysis.

**Results:**

SAE accounted for about 50% in patients with sepsis and was independently associated with the 30-day mortality of sepsis. Variables eligible for the nomogram included patient’s age and clinical parameters on the first day of ICU admission including the GCS score, lactate, bilirubin, red blood cell distribution width (RDW), mean value of respiratory rate and temperature, and the use of vasopressor. Compared with Sequential Organ Failure Assessment (SOFA) and Logistic Organ Dysfunction System (LODS), the nomogram exhibited better discrimination with an area under the receiver operating characteristic curve (AUROC) of 0.763 (95%CI 0.736–0.791, *p* < 0.001) and 0.753 (95%CI 0.713–0.794, *p* < 0.001) in the training and validation sets, respectively. The calibration plot revealed an adequate fit of the nomogram for predicting the risk of 30-day mortality in both sets. Regarding to clinical usefulness, the DCA of the nomogram exhibited greater net benefit than SOFA and LODS in both of the training and validation sets. Besides, the nomogram exhibited acceptable discrimination, calibration, and clinical usefulness in sensitivity analysis.

**Conclusions:**

SAE is related to increased 30-day mortality of patients with sepsis. The nomogram presents excellent performance in predicting 30-day risk of mortality in SAE patients, which can be used to evaluate the prognosis of patients with SAE and may be more beneficial once specific treatments towards SAE are developed.

## Introduction

Sepsis-associated encephalopathy (SAE) is the dysfunction of brain that develops during the process of sepsis without evidence of the central nervous system (CNS) infection. It is tightly associated with long-term impairment of behavior, memory, and cognitive function, imposing heavy medical and financial burden on families and society [1–3]. More harmfully, patients with SAE tend to have higher short-term mortality than those with sepsis alone. A landmark study conducted by LA et al. demonstrated that encephalopathy is associated with increased hospital mortality from 16% when the Glasgow Coma Score (GCS) is 15 to 63% when GCS is between 3 and 8 [4]. Similar conclusions were drew from another high-quality study performed by Sonneville et al., which showed decreased 30-day survival rate from 67% when GCS is 15 to 32% when GCS is between 3 and 8, and even mild change in consciousness (defined by GCS of 13–14) is an independent risk factor for the 30-day mortality with an hazard rate (HR) of 1.38 after adjusting for confounding factors [5]. Except for these two high quality studies, several other researches also reported that SAE was responsible for increased short-term mortality, prolonged hospitalization time, or overmuch assumption of medical resources [6, 7]. Based on these, identifying SAE patients with high risk of short-term mortality is of great significance for that it may facilitates timely medical intervention and improves the prognosis of such patients. Therefore, the main objective of the present study by a large clinical database is to evaluate the impact of SAE on the 30-day mortality of patients with sepsis and then develop a predictive nomogram to individually predict the probability of 30-day death in SAE patients.

## Material and methods

### Data source

We conducted an observational study by retrieving data from the Medical Information Mart for Intensive Care (MIMIC III) open source clinical database, which contains de-identified health-related data of over forty thousand patients who received treatment in critical care units of the Beth Israel Deaconess Medical Center between June 2001 and October 2012 [8, 9]. The database is continuously updated, and the newest version (MIMIC-III v1.4) was released on 2 September 2016, which enhanced data quality and provided a large amount of additional data. We use the MIMIC-III v1.4 in our study, and all data in it was classified into 26 tables recording various individual information, such as demographic characteristics, treatment measures, nursing notes, and laboratory tests. Besides, it contains prognostic data obtained from the hospital and laboratory health record systems reporting the hospital mortality, or from the Social Security Administration Death Master File recording the out-of-hospital survival data. The MIMIC III database can be freely utilized after successful application and ethical approval from the Institutional Review Boards of both Beth Israel Deaconess Medical Center (Boston, MA, USA) and the Massachusetts Institute of Technology (Cambridge, MA, USA). Since all data are de-identified in this database to remove patients’ information, the requirement for individual patient consent is not indispensable.

### Study population and data extraction

PgAdmin (version 4.1, Bedford, USA) was used to run structure query language (SQL) and then to extract data from the MIMIC III database. Six tables were occupied in our study, including DIAGNOSES_ICD, ICUSTAYS, PATIENTS, LABEVENTS, MICROBIOLOGYEVENTS, and PRESCRIPTIONS. We included adult patients (> 17 years of age) with a diagnosis of sepsis according to the Third International Consensus Definitions for Sepsis (Sepsis-3): (1) Patients with infection confirmed by the positive results of microbial cultivation and (2) the Sequential Organ Failure Assessment (SOFA) score ≥ 2 [10]**.** Excluded were patients (1) with primary brain injury (traumatic brain injury, ischemic stroke, hemorrhagic stroke, epilepsy, or intracranial infection); (2) with pre-existing liver or kidney failure affecting consciousness; (3) with severe burn and trauma; (4) receiving cardiac resuscitation recently; (5) with chronic alcohol or drug abuse; (6 )with severe electrolyte imbalances or blood glucose disturbances, including hyponatremia (< 120 mmol/l), hyperglycemia (> 180 mg/dl), or hypoglycemia (< 54 mg/dl); (7) dying or leaving within 24 h since ICU admission; and (8) without an evaluation of GCS. Eligible patients were included into the final cohort for investigation. For the final cohort, we retrospectively collected the following data: (1) demographic data including age, gender, and ethnicity; (2) 30-day mortality, in which no patient was lost to follow-up during 30 days, and patients live longer than 30 days were recorded as survival; (3) comorbidity as coded and defined in the International Classification of Diseases, Ninth Revision (ICD-9); (4) mean value of vital signs during the first 24 h of ICU stay; (5) the first laboratory data since ICU admission; and (6) site of infection and type of micro-organism. The severity of illness and organ failure was assessed by modified forms of the simplified acute physiology score (SAPSII) and sepsis-related organ failure assessment (SOFA), respectively, on the first day of ICU admission [11, 12]. The modified forms are SAPSIIand SOFA excluding the component of central nervous system. Besides, we created another dataset for sensitivity analysis based on the Martin’s criteria, a widely used approach for identifying sepsis in administrative health data [13] (Additional file 1: Figure S1).

### Sepsis-associated encephalopathy

We defined SAE in the study as sepsis accompanied by GCS ≤ 14 on the first day of ICU admission or delirium according to the ICD-9 code (2930, 2931). The delirium caused by alcohol or drug abuse, dimension, mental disorders, and neurological diseases was excluded from the definition of SAE. GCS was confirmed as an excellent tool for characterizing SAE and distinguishing it from sepsis [4] and for neurological evaluation of critically ill patients [14]. For sedated or postoperative patients, we adopted GCS measured before sedation or surgery.

### Statistical analysis

Shapiro-Wilk tests were used to assess the distribution of variables. Data were expressed as mean ± standard deviation (SD) for parametric continuous data and as median (interquartile ranges) for non-parametric distribution. Categorical data was expressed as number (percentages). Parametric continuous variables were compared by using unpaired Student’ *t* test and non-parametric continuous variables by Mann–Whitney *U* test. The chi-squared test was adopted to assess the differences in categorical variables between groups.

Logistic regression analysis was used to identify risk factors independently associated with the 30-day mortality of sepsis. Specifically, variables related to 30-day death in univariate analysis (*p* < 0.1) were entered into multivariate logistic regression analysis to calculate estimated odds ratios (OR) and 95% confidence intervals (95%CI), where significant level for independent risk factors was *p* < 0.05. Collinearity between continuous variables was tested by the variance inflation factor (VIF), and an arithmetic square root of VIF ≤ 2 was considered as non-collinearity. Kaplan–Meier analysis was conducted to visualize the probability of 30-day survival between SAE and non-SAE cohorts, and log-rank test was used to identify between-group difference. Besides, propensity score match (PSM) was conducted between the SAE and non-SAE cohorts, and then the 30-day survival was visualized by Kaplan–Meier analysis and compared by log-rank test.

In the process of nomogram development, patients with SAE were randomly distributed into a training set and a validation set without replacement at a ratio of 7:3. Logistic regression analysis following the steps mentioned above was conducted to identify independent risk factors for the 30-day mortality of SAE. Then, a nomogram in predicting the probability of 30-day death was obtained by the training set according to Occam’s Law of Razor, namely, the best model should be one that can achieve the aim of study with fewer variables [15]. The performance of the nomogram was evaluated and compared with SOFA and Logistic Organ Dysfunction System (LODS) [16] in both of the training and validation sets by an area under the curve of the receiver operating characteristic (AUROC) and by calibration with bootstrap method with 1000 resampling. Besides, integrated discrimination improvement (IDI) was calculated to compare discrimination slopes and Brier score to evaluate model fitness [17]. DCA analysis was performed to evaluate the net benefit of medical intervention conforming nomogram, SOFA, and LODS at different threshold probabilities in the training and validation sets. Sensitivity analyses were conducted in two cohorts, namely, sensitivity-1 in the cohort diagnosed by Martin’s criteria and sensitivity-2 in a subset (GCS 3-8) of the cohort diagnosed by Sepsis-3.

Statistical analyses were performed using the R software (version 3.6.1, R Foundation for Statistical Computing, Vienna, Austria). Missing values were addressed with multiple imputation in the process of logistic regression and model construction. The imputation technique involves creating multiple copies of the data and replacing missing values with imputed values through a suitable random sample from their predicted distribution. We used the “mice” package of R to obtain 5 imputation datasets. A two-tailed *p* value of < 0.05 was considered statistically significant. All analyses were reported according to the Transparent Reporting of a Multivariable Prediction Model for Individual Prognosis or Diagnosis (TRIPOD) guidelines [18].

## Results

### Characteristics of participants with sepsis

After screening by the inclusion and exclusion criteria, a total of 4987 patients were included into the final cohort, and SAE was observed in 2474 (49.6%) patients. Characteristics at baseline and upon ICU admission of all participants and participants in SAE and non-SAE groups were exhibited in Tables 1 and 2. Patients with SAE were older than those without it, with a median age of 73 [58, 83] in SAE patients and 68 [55, 79] in non-SAE ones. Patients with SAE were more likely to admit into the medical intensive care unit (MICU) and suffer from hypertension, anemias, and history of neurological diseases, which mainly included Parkinson’s disease, Huntington’s chorea, and leukodystrophy. Besides, patients with SAE had lower level of partial pressure of blood oxygen (PO2), bilirubin, lactate, prothrombin time (PT), and higher level of serium PH value on the first day of ICU admission. The 30-day mortality of SAE and non-SAE cohorts was 527 (21.30%) and 449 (17.87%), respectively, with a statistical difference of *p* = 0.003. Interestingly, except for CNS, the severity of illness and organ failure was more serious in non-SAE patients than that in SAE ones, with *p* < 0.001 in both of the modified SAPSIIand SOFA. Besides, patients without SAE exhibited higher frequent use of vasopressor and longer hospital and ICU stay time in our study.

**Table 1:** Patients’ baseline characteristics

Variable	All patients*, n* = 4987	Non-SAE patients*, n* = 2513	SAE patients*, n* = 2474	*P* value
Age, years	70 [56, 81]	68 [55, 79]	73 [58, 83]	< 0.001
Gender, male	2680 (53.74)	1434 (57.06)	1246 (50.36)	< 0.001
**Ethnicity,*n*(%)**				0.002
White	3700 (74.19)	1845 (73.42)	1855 (74.98)	
Black	380 (7.62)	166 (6.61)	214 (8.65)	
Hispanic or Latino	145 (2.91)	78 (3.10)	67 (2.71)	
Asian	106 (2.13)	56 (2.23)	50 (2.02)	
Others	656 (13.15)	368 (14.64)	288 (11.64)	
**Comorbidity,*n*(%)**				
Cardiovascular diseases	2772 (55.58)	1413 (56.23)	1359 (54.93)	0.372
Peripheral vascular disease	552 (11.07)	299 (11.90)	253 (10.23)	0.066
Other neurological diseases	683 (13.70)	189 (7.52)	494 (19.97)	< 0.001
Hypertension	2457 (49.27)	1181 (47.00)	1276 (51.58)	0.001
Chronic pulmonary disease	1136 (22.78)	593 (23.60)	543 (21.95)	0.176
Diabetes	909 (18.23)	449 (17.87)	460 (18.59)	0.530
Hypothyroidism	579 (11.61)	272 (10.82)	307 (12.41)	0.089
Liver disease	421 (8.44)	215 (8.56)	206 (8.33)	0.811
Coagulopathy	880 (17.65)	476 (18.94)	404 (16.33)	0.017
Anemias	1303 (26.13)	625 (24.87)	678 (27.41)	0.045

Continuous data are presented as median (interquartile range), whereas categorical data are presented as frequency (percentage)

**Table 2:** Patients’ characteristics at ICU admission

Variable	All patients, *n* = 4987	Non-SAE patients, *n* = 2513	SAE patients, *n* = 2474	*P* value
Hospital stay time, days	11.50 [6.80, 19.90]	11.90 [7.10, 20.20]	10.95 [6.40, 19.60]	< 0.001
ICU stay time, days	3.50 [1.90, 7.70]	3.90 [2.00, 9.00]	3.00[1.80, 6.30]	< 0.001
30-day mortality, *n* (%)	976 (19.57)	449 (17.87)	527 (21.30)	0.003
Mechanical ventilation, *n* (%)	437 (8.76)	228 (9.07)	209 (8.45)	0.465
Vasopressor	1476 (29.60)	899 (35.77)	577 (23.32)	< 0.001
**First care unit,*n*(%)**				< 0.001
CCU	576 (11.55)	343 (13.65)	233 (9.42)	
CSRU	576 (11.55)	353 (14.05)	223 (9.01)	
MICU	2459 (49.31)	1164 (46.32)	1295 (52.34)	
SICU	773 (15.50)	360 (14.33)	413 (16.69)	
TSICU	603 (12.09)	293 (11.66)	310 (12.53)	
**Severe Score**^**b**^				
Modified SOFA	4.60 ± 2.87	5.21 ± 2.77	3.98 ± 2.84	< 0.001
Modified SAPSII	38.54 ± 12.04	39.63 ± 11.99	37.44 ± 11.99	< 0.001
**Vital signs**^**c**^				
Mean heartrate (min^−1^)	89.27 ± 16.33	89.29 ± 16.12	89.25 ± 16.54	0.933
Mean arterial pressure (mmHg)	74.46 ± 9.91	73.31 ± 8.66	75.63 ± 10.91	< 0.001
Mean respiratory rate (min^−1^)	20.08 ± 4.15	20.08 ± 4.00	20.07 ± 4.31	0.948
Mean temperature (°C)	36.93 ± 0.67	36.98 ± 0.69	36.87 ± 0.66	< 0.001
Mean SpO2 (%)	97.3 [96.0, 98.5]	97.3 [96.0, 98.5]	97.3 [96.0, 98.5]	0.092
**Laboratory tests**^**d**^				
Lactate (mmol/L)	1.6 [1.1, 2.4]	1.6 [1.1, 2.5]	1.5 [1.1, 2.2]	< 0.001
PCO_2_ (mmHg)	40 [35.0, 46.5]	40 [35,46]	40 [35,47]	0.898
PO_2_ (mmHg)	110 [75, 195]	117 [77, 217]	103 [73, 175]	< 0.001
PH	7.371 ± 0.095	7.364 ± 0.098	7.378 ± 0.092	< 0.001
Creatinine (K/uL)	1.1 [0.8, 1.7]	1.1 [0.8, 1.7]	1.1 [0.8, 1.6]	0.002
BUN (K/uL)	23 [15, 37]	23 [16, 37]	23 [15, 38]	0.087
ALT^**e**^	1.4 [1.2, 1.7]	1.4 [1.2, 1.7]	1.4 [1.2, 1.7]	0.224
AST^**f**^	1.5 [1.3, 1.8]	1.5 [1.3, 1.8]	1.5 [1.3, 1.8]	0.208
Bilirubin (EU/dL)	0.6 [0.4, 1.2]	0.7 [0.4, 1.3]	0.6 [0.4, 1.1]	< 0.001
Hemoglobin (g/dL)	11.49 ± 2.23	11.50 ± 2.27	11.49 ± 2.19	0.890
Platelet (K/uL)	227 [159.5, 306.0]	220 [154, 304]	232 [167, 308]	0.003
Potassium (K/uL)	4.2 [3.8, 4.6]	4.1 [3.8, 4.6]	4.2 [3.8, 4.7]	0.208
Sodium (K/uL)	138 [135, 141]	138 [135, 141]	138 [135, 141]	0.687
PT (s)	13.9 [12.9,16.2]	14.1 [13.0,16.3]	13.8 [12.8,16.0]	< 0.001
RDW (%)	14.8 [13.7, 16.4]	14.8 [13.7, 16.5]	14.7 [13.7, 16.4]	0.359
WBC (K/uL)	10.70 [7.30, 15.25]	10.60 [7.30, 15.30]	10.80 [7.30, 11.50]	0.751
Lymphocyte (%)	9.4 [5.6, 16.0]	9.1 [5.4, 15.9]	9.6 [5.8, 16.3]	0.086
Neutrophil (%)	81.2 [72, 88]	81.3 [71.9, 88.0]	81.15 [72.13, 88.00]	0.342
MCV (fL)	90 [86, 95]	90 [86, 94]	91 [86,95]	0.002
**Infection site,*n*(%)**				
Urine	2221 (44.54)	997 (39.67)	1224 (49.47)	< 0.001
Blood	1319 (26.45)	679 (27.02)	640 (25.87)	0.374
Lung	1915 (38.40)	1090 (43.37)	825 (33.35)	< 0.001
Catheter	249 (4.99)	135 (5.37)	114 (4.61)	0.241
Gastrointestinal tract	302 (6.06)	148 (5.89)	154 (6.22)	0.662
Abdominal cavity	113 (2.27)	58 (2.31)	55 (2.22)	0.914
Skin/soft tissue	828 (16.60)	404 (16.08)	424 (17.14)	0.332
Others	170 (3.41)	102 (4.06)	68 (2.75)	0.013
**Microorganisms,*n*(%)**				
Gram-positive	2502 (50.17)	1237 (49.22)	1265 (51.13)	0.187
Gram-negative	1986 (39.82)	1002 (39.87)	984 (39.77)	0.966
Fungus	1522 (30.52)	823 (32.75)	699 (28.25)	< 0.001

Parametric continuous data are presented as mean ± standard deviation (SD), and non-parametric continuous data are presented as median (interquartile ranges), whereas categorical data are presented as frequency (percentage)

^b^Severe score is calculated on the first day of each ICU patients’ stay

^c^Vital signs are calculated on the first 24 h of each ICU patients’ stay

^d^Laboratory tests recorded the first result of each patients’ ICU stay

^e^ALT in the table is the value after logarithmic transformation

^f^ALT in the table is the value after logarithmic transformation

*CCU* coronary care unit, *CSRU* cardiac surgical intensive care unit, *MICU* medical intensive care unit, *SICU* surgical intensive care unit, *TSICU* trauma/surgical intensive care unit, *SOFA* Sequential Organ Failure Assessment, *SAPSII* the simplified acute physiology score, *RDW* red blood cell distribution widths, *MCV* mean corpuscular volume

### SAE is independently associated with the 30-day mortality of patients with sepsis

After adjusting for baseline characteristics, vital signs, laboratory tests, infection site, and microorganisms, the results of multivariate logistic regression revealed that SAE was an independent risk factor for the 30-day mortality of patients with sepsis [adjusted odd ratio (aOR) = 1.26, 95% CI 1.07–1.49, *p* = 0.005] (Additional file 2:Table S1). Kaplan–Meier’s survival estimates of patients according to the presence or absence of SAE at ICU admission were presented in Additional file 3: Figure S2. After conducting propensity score match (PSM) between SAE and non-SAE groups according to the differences in baseline characteristics and characteristics at ICU admission (Additional file 4: Figure S3), Kaplan–Meier’s survival analysis was conducted, and results were presented in Additional file 5: Figure S4. Results showed that no matter performing PSM or not, significant differences were observed in 30-day survival between SAE and non-SAE patients, with log-rank *p* = 0.0018 before PSM and log-rank *p* < 0.0001 after PSM.

### Development of a prediction nomogram in the training set

Characteristics at baseline and upon ICU admission of SAE patients and participants in the training set and validation set were exhibited in Additional file 6: Table S2 and Additional file 7: Table S3. Results revealed that both sets had no statistic difference in all the variables.

**Fig. 1:**
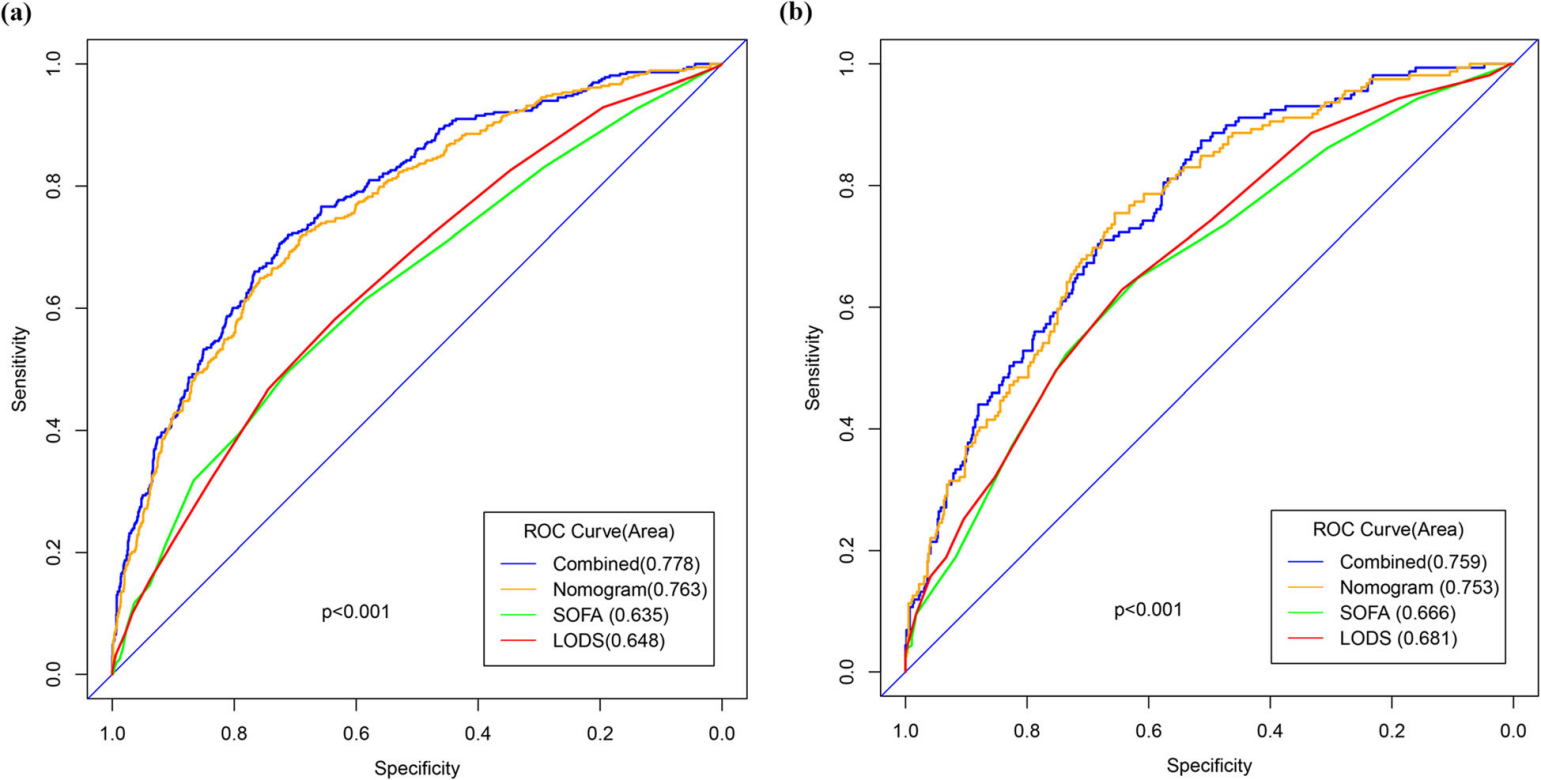
The ROC curve of the combined model, the prediction nomogram, SOFA, and LODS in the training set (**a**) and validation set (**b**). The combined model is incorporated by all the independent risk variables. The prediction nomogram includes age, lactate, bilirubin, RDW, mean value of respiratory rate and temperature, and the use of vasopressor

**Fig. 2:**
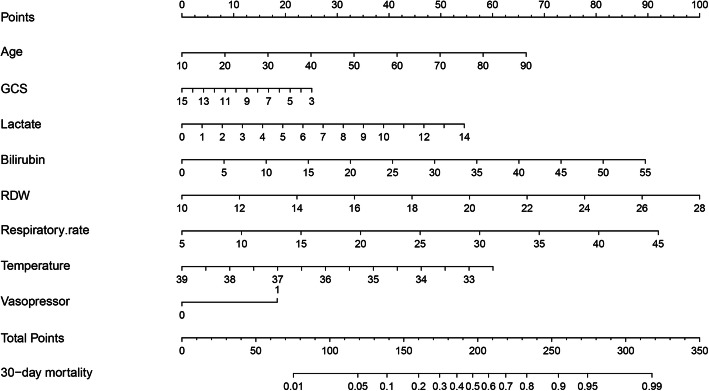
Validated nomogram for predicting 30-day mortality of SAE. When using it, drawing a vertical line from each variables upward to the points and then recording the corresponding points (i.e., “RDW = 14” = 20 points). The point of each variable was then summed up to obtain a total score that corresponds to a predicted probability of 30-day death at the bottom of the nomogram

The risk factors related to 30-day mortality of SAE identified by multivariable logistic regression were shown in Table 3. The VIF was calculated, and no continuous variables in Table 3 had an arithmetic square root of VIF ≤ 2. Furthermore, the correlation between continuous variables was visualized in Additional file 8: Figure S5, and linear correlation was not observed, indicating that collinearity was not existed in the regression model. Lung infection and catheter-related infection were excluded from model development since these two parameters depended on microbial culture, which is a time-consuming process. The performances of the remaining risk factors in Table 3 were then comprehensively evaluated and finally, according to the Occam’s Law of Razor, a model integrating age, lactate, bilirubin, RDW, mean value of respiratory rate and temperature, and the use of vasopressor was established for its similar discrimination compared with the model including all the risk factors (combined) in both of the training and validation sets (Fig. 1). Based on this model, a nomogram was plotted to predict the probability of 30-day death of SAE (Fig. 2).

**Table 3:** Factors independently associated with 30-day mortality of patients with SAE in the multivariate logistic analysis

Variables	OR	95% CI		*P* value
Age (year)	1.04	1.03	1.05	< 0.001
GCS score	0.91	0.87	0.95	< 0.001
Lactate (mmol/L)	1.19	1.08	1.31	< 0.001
PO_2_ (mmHg)	1.00	1.00	1.00	0.023
Sodium (K/uL)	0.97	0.94	0.99	0.009
Bilirubin (EU/dL)	1.05	1.01	1.10	0.013
RDW (%)	1.22	1.14	1.30	< 0.001
MCV (fL)	1.02	1.01	1.04	0.007
Mean heart rate (min-1)	1.01	1.00	1.02	0.019
Mean respiratory rate (min-1)	1.06	1.03	1.10	< 0.001
Mean temperature (°C)	0.69	0.55	0.86	< 0.001
Mean SpO2 (%)	0.92	0.87	0.98	0.008
Catheter-related infection	0.33	0.15	0.66	0.003
Lung infection	1.76	1.29	2.39	< 0.001
Vasopressor	1.59	1.18	2.15	0.003

*GCS* Glasgow Coma Score, *RDW* red blood cell distribution widths, *MCV* mean corpuscular volume

Hosmer–Lemeshow test: *p* = 0.834

### Validation of the prediction nomogram

We compared the nomogram with SOFA and LODS for predicting 30-day risk of mortality in SAE patients, and results were shown in Table 4. Results showed that the AUROC of the nomogram was significantly higher than that of SOFA and LODS in both of the training and validation sets, indicating that the predictive nomogram had better discrimination than SOFA and LODS in predicting the 30-day mortality of patients with SAE.

**Table 4:** Comparison of models in predicting the 30-day mortality of SAE

Predictive Model		AUROC	*P* value	IDI	*P* value	Brier index	*P* value
**Training set**	Nomogram	0.763 [0.736–0.791]				0.139 [0.129–0.150]	
	SOFA	0.635 [0.602–0.667]	< 0.001	0.126[0.106–0.145]	< 0.001	0.161 [0.150–0.171]	< 0.001
	LODS	0.648 [0.617–0.679]	< 0.001	0.119[0.098–0.140]	< 0.001	0.159 [0.149–0.170]	< 0.001
**Validation set**	Nomogram	0.753 [0.713–0.794]				0.144 [0.128–0.159]	
	SOFA	0.666 [0.619–0.713]	< 0.001	0.082[0.054–0.110]	< 0.001	0.157 [0.141–0.174]	< 0.001
	LODS	0.681 [0.635–0.727]	< 0.001	0.071[0.041–0.101]	< 0.001	0.155 [0.139–0.172]	< 0.001

The *P* value was drew by comparing the results of nomogram with SOFA or LODS

*SOFA* Sequential Organ Failure Assessment, *AUROC* area under the receiver operating characteristic curve, *IDI* integrated discrimination improvement

**Fig. 3:**
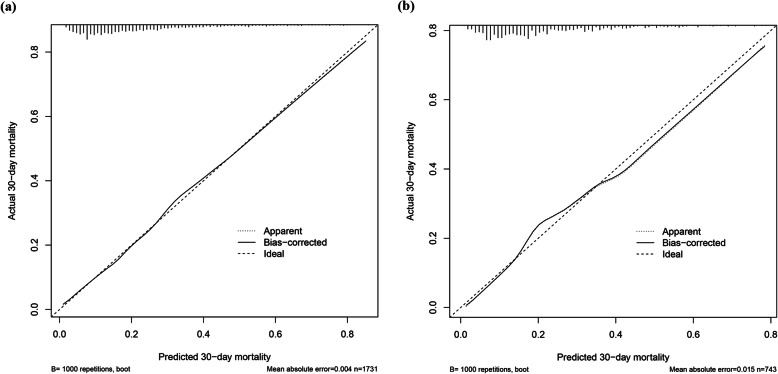
Calibration curves constructed by bootstrap approach in the training set (**a**) and validation set (**b**). In both sets, the apparent curve and bias-corrected curve slightly deviated from reference line, but a good conformity between observation and prediction is observed

Calibration curves were depicted for both of the training and validation sets, and the bias-corrected line is formed using a bootstrap method. In both sets, the apparent curve and bias-corrected curve slightly deviated from reference line, but a good conformity between observation and prediction is still observed (Fig. 3). The Brier score of the nomogram was 0.139 (95%CI 0.129–0.150) in the training set and 0.144 (95%CI 0.128–0.159) in the validation set, which were higher than that of SOFA and LODS in both sets, indicating that the nomogram had better calibration of prediction than SOFA and LODS. Moreover, the IDI of the nomogram was significantly higher than that of SOFA and LODS in both sets, revealing that the nomogram could increase the prediction probability of SOFA and LODS in the two sets.

### Clinical use of the nomogram

**Fig. 4:**
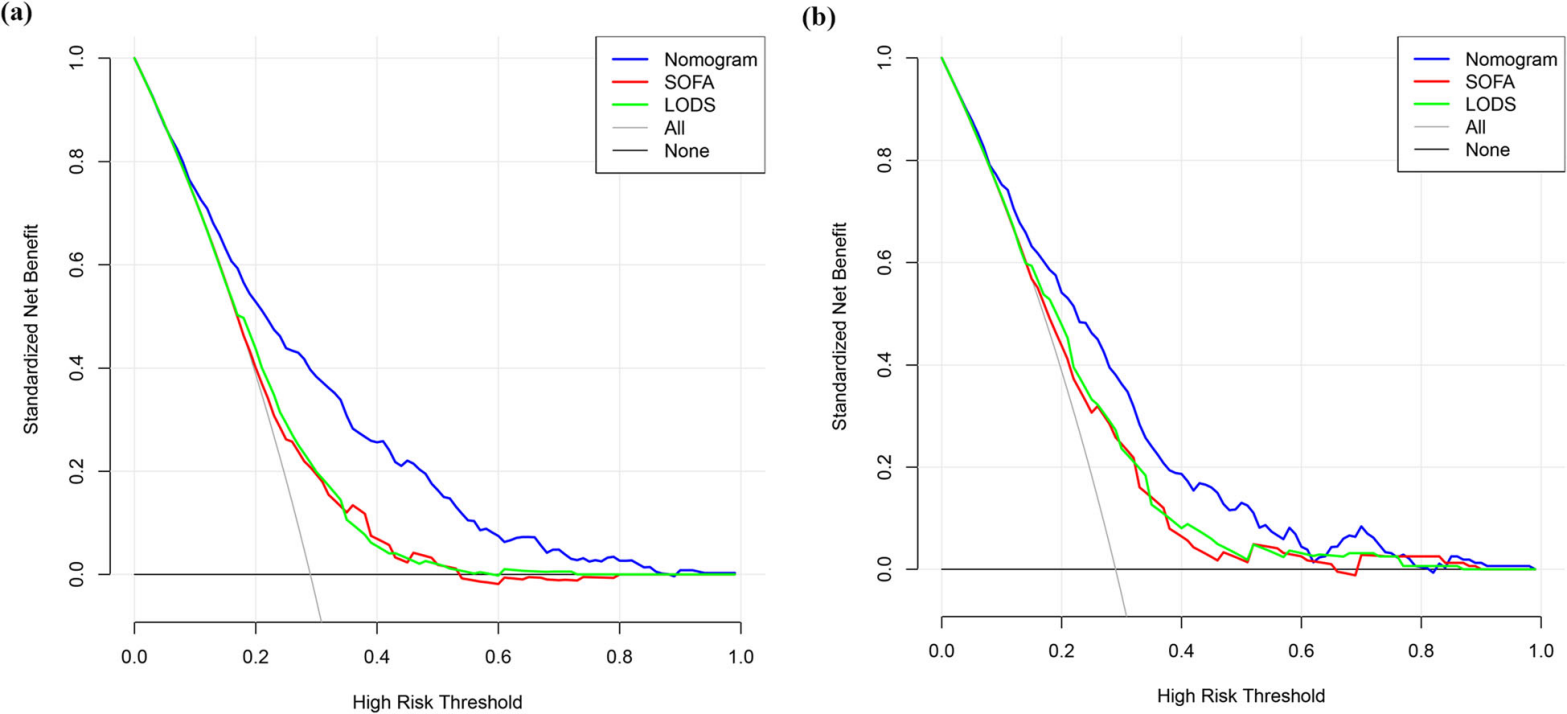
The DCA curve of medical intervention in patients with the nomogram, SOFA, and LODS in the training set (**a**) and validation set (**b**)

With regard to clinical use, the DCA for nomogram was depicted and compared with SOFA and LODS. In the training set, medical intervention guided by the nomogram could add more net benefit than SOFA and LODS when the threshold probability (PT) > 0.1 (Fig. 4a). In the validation set, treatment directed by nomogram could gain more net benefit than SOFA and LODS when PT was between 0.1 and 0.6 (Fig. 4b).

### Sensitivity analysis

We conducted sensitivity analysis by including patients with SAE diagnosed by the Martin’s criteria (sensitivity-1) or patients with GCS of 3-8 in the dataset extracted by Sepsis-3 (sensitivity-2). The discrimination, calibration, and clinical usefulness of the nomogram were compared with SOFA and LODS by using AUROC, calibration curve, DCA curve, Brier index, and IDI (Additional file 9: Figure S6; Additional file 10: Figure S7; Additional file 11: Table S4). Results showed that the prediction nomogram had better discrimination and calibration as well as more clinical net benefit than SOFA and LODS in both of the sensitivity analyses, which further consolidated the stable and excellent predictive performance of the nomogram.

## Discussion

In this retrospective analysis by the MIMIC III database, we conducted logistic regression to recognize the risk factors related to the 30-day mortality of patients with SAE and predictors including age, lactate, bilirubin, RDW, mean value of respiratory rate and temperature, and the use of vasopressor that were identified and integrated into a best-fit prediction model visualized as a prediction nomogram. To the best of our knowledge, this is the first study to evaluate the independent risk factors associated with the 30-day mortality of SAE and develop a nomogram to predict it.

EW et al. find that 20–50% of patients with sepsis have delirium or impairment of consciousness, indicating that SAE is highly prevalent in the ICU and regraded to be the most common encephalopathy in the surgical intensive care unit [19]**.** CL et al. found that patients with acutely altered mental status associated with sepsis have higher mortality rates (49%) than septic patients with pre-existing mental status changes (41%) or normal mental status (26%) [6]. Since then, several other studies conducted in different study cohorts further supported their conclusion that SAE is closely related to the increased short-term mortality of patients with sepsis [4, 5, 20]. Based on these, two limitations seem obvious and need to be solved urgently. One is that treatment towards SAE is still challenging by the fact that the widely used guidelines of sepsis listed a variety of evidence-based recommendations for the treatment of sepsis, but none for SAE [21–23]. This predicament may reveal another limitation that clinicians do not pay enough attention to SAE, which can be reflected by the fact that they seem to be overly optimistic about prognosis and the therapeutic impact of treatment in patients with sepsis [24–28]. Therefore, it is important for clinicians to comprehensively evaluate the true risks of mortality and objectively estimate the risks/benefits of medical intervention in patients with sepsis to allow clinicians, patients, and their families the ability to carefully evaluate the impact of potential treatment options, help them to make medical decision together, and prevent medical disputes. The prediction nomograms is therefore essential to an improved risk stratification process of sepsis and then useful to a clear statement of the condition by clinicians to the families of patients with SAE. Further studies should focus on the pathogenesis of SAE and the development of specific treatment to it, which may further enhance the clinical usefulness of the nomogram by reducing the mortality of SAE. As assessment with a nomogram may be time consuming and complicated to use in clinical practice, developing a software which can be embedded into the electronic medical system is our next work, which can guide clinicians for the timely treatment of SAE and reduce the mortality of patients without increasing the working time and burden of clinicians.

SOFA and LODS had been demonstrated to be useful tools in predicting the short-term mortality of patients with sepsis [10, 29], but whether they are applicable to SAE is still unclear. Thus, we developed the current predictive model in the datasets extracted by Sepsis-3 and compared its predictive performance with SOFA and LODS in both of the datasets extracted by Sepsis-3 and by the Martin’s criteria. Results showed that SOFA and LODS in discriminating SAE patients under the risk of 30-day death were not so good as the results reported in patients with sepsis in the previous studies, whereas the prediction nomogram could improve the predictive performance of SOFA and LODS and exhibited acceptable discrimination and calibration. Besides, to validate its clinical usefulness, decision curve analysis was employed to account for both the benefits and the costs of medical intervention to SAE patient guided by the nomogram. Results revealed that interventions guided by the current nomogram can add more net benefits than SOFA and LODS.

RDW accounted for the biggest weight in the nomogram, indicating that it is the most important predictor and has the strongest power to predict 30-day mortality of SAE patients. RDW is a measure of the size of circulating erythrocytes and was routinely used in the differential diagnosis of anemia. However, studies have revealed that it is also useful in estimating the short-term mortality of non-hematologic diseases, such as cardiovascular diseases [30, 31], stroke [32], liver diseases [33, 34], and critical illness [35]. Consistently, our study demonstrated that RDW is an independent risk factor and potent predictor for the 30-day mortality of SAE. The relationship between RDW and the 30-day mortality of SAE was shown in Additional file 12: Figure S8, indicating that the 30-day mortality of SAE was positively associated with the level of RDW. Mechanisms under the relationship between RDW and the 30-day mortality of SAE remain largely unknown, but several studies had revealed that the inflammatory response during sepsis may contribute to the adverse impact of RDW on the prognosis of SAE as RDW is positively associated with inflammatory markers, such as C-reactive protein (CRP) and erythrocyte sedimentation rate (ESR) [36–39]. Besides, oxidative stress may be another reason to connect RDW with poor 30-day outcome because studies indicated that oxidative stress can increase anisocytosis by disrupting erythropoiesis and altering the circulating half-life of red blood cell, ultimately leading to increased level of RDW [40, 41].

The diagnosis of sepsis remains controversial for that the pathophysiology of sepsis is still not fully understood. Both of Sepsis-3 and the systemic inflammatory response syndrome (SIRS) definitions are currently used in clinical practice to diagnose patients with sepsis [10, 12], but each has its own defects. For Sepsis-3, the parameters needed in SOFA are burdensome to collect and not usually available at the bedside to help with clinical decision-making. qSOFA is simpler, but it was only validated outside the ICU and not suitable to patients which already admitted to an ICU. Besides, Sepsis-3 relies on the clinician’s experience to identify suspected infection, which may not be apparent early on, making it less sensitive than SIRS for diagnosing early sepsis [42]. SIRS definition based on SIRS to diagnose sepsis, but changes in white blood cell count, temperature, and heart rate only reflect inflammation, the more dangerous host response to infection cannot be well indicated by the criteria of SIRS, and studies has demonstrated that SIRS criteria are present in many hospitalized patients, including those who never develop infection and never incur adverse outcomes [43]. Therefore, we established two datasets in this study by extracting data from patients with sepsis diagnosed by the Sepsis-3 criteria (dataset-1) and Martin’s criteria (dataset-2), respectively. Then, we developed the prediction nomogram in dataset-1 and conducted sensitivity analysis in dataset-2. Results showed that the nomogram exhibited stable and acceptable predictive performance in both of the two datasets. However, we are still cautious about the prediction efficiency of the nomogram in the condition of Sepsis-3. In Sepsis-3 [10, 29], infection is identified as patients who had body fluids sampled for culture and received antibiotics, which required the combination of culture and antibiotic start time to occur within a specific time epoch, namely, if the antibiotic was given first, the culture sampling must have been obtained within 24 h. If the culture sampling was first, the antibiotic must have been ordered within 72 h, but it is difficult to extract data like this in our study as culture sampling time is lacking in the MIMIC III database. Consequently, we just include patients with infection confirmed by the positive results of microbial cultivation, which may result in the fact that the dataset of our study is smaller than that extracted by the definition of Sepsis-3. Therefore, further studies based on our own data strictly extracted by Sepsis-3 should be performed to further validate the predictive performance of the nomogram in the condition of Sepsis-3.

Two points should be noted when using the nomogram. First, as vital signs in our study are the mean values of the first 24 h of each ICU patient, and the nomogram is not applicable to patients dying or leaving within 24 h since ICU admission. Second, laboratory tests in the nomogram are the first results since ICU admission; therefore, all the laboratory tests included in the nomogram should be completed within the first 24 h since ICU admission.

This study has some limitations: First, one of the challenges in studying SAE is that without specific diagnostic method, it remains a rule-out definition, which may lead to a high specificity, but relatively low sensitivity for the diagnosis of SAE. Thus, the current nomogram can only be used in SAE diagnosed by exclusion and may require further modification once specific diagnostic methods are developed. Second, the retrospective nature of this observational study determined that unidentified confounding factors may affect the results if adding to the model. Third, studies based on the results of brain MRI have revealed that the impairment of cerebral white matter in patients with critical illness are not only related to sequelae of the central nervous system but also associated with increased mortality [44], but neuroimaging data was not included in the study, making it impossible to assess the impact of organic lesion of brain on outcome of SAE. Finally, as data in the MIMICIII database is slightly old and we only conducted an internal validation by this database, external validation based on our own data should be performed in the future study to further validate the robustness and performance of the nomogram.

## Conclusion

A prediction nomogram based on patients’ age, together with the GCS score, lactate, bilirubin, RDW, mean value of respiratory rate and temperature, and the use of vasopressor on the first day of ICU admission can be conveniently used to serve accurate prognostic prediction in the 30-day mortality of SAE. This may be particularly beneficial in preventing the deterioration of SAE once specific treatments towards encephalopathy are developed and finally improve the prognosis of SAE patients.

## Supplementary information

**Additional file 1: Figure S1.** Flowchart of data extraction. Patients with sepsis were extracted from the MIMIC III database by both of Sepsis-3 and the Martin’s criteria. Then, we excluded patients with comorbidities that may have adverse impact on consciousness, or without a record of GCS, or died within 24 hours since ICU admission. The order of exclusion was consistent with what we performed by the SQL. After exclusion , patients remained in the “ Sepsis-3” cohort (blue) were picked out to make up the final cohort and those with GCS 3-8 were used to develop the nomogram and conducted sensitivity analysis, respectively. Besides, patients with SAE in the “Martin’s criteria” cohort (red) were picked out to conduct sensitivity analysis.

**Additional file 2: Table S1.** Factors independently associated with 30-day mortality of patients with sepsis in the multivariate logistic analysis.

**Additional file 3: Figure S2.** The Kaplan-Meier’s survival estimated of the 30-day survival probability of SAE and non-SAE patients. Results showed that the 30-day survival of SAE patients were significantly lower than that of non-SAE patients (Log-rank p = 0.0018).

**Additional file 4: Figure S3.** Propensity score match (PSM) between SAE and non-SAE patients. The statistically different variables in baseline characteristics and characteristics at ICU admission of patients with sepsis were exhibited as the red dot (unmatched) and all of them were matched well and similar between the two groups (green dot: matched) after PSM.

**Additional file 5: Figure S4.** The Kaplan-Meier’s survival estimated of the 30-day survival probability of SAE and non-SAE patients after PSM. Results showed that the 30-day survival of SAE patients were significantly lower than that of non-SAE patients (Log-rank p < 0.0001).

**Additional file 6: Table S2.** Baseline characteristics of patients in the training and validation sets^a^.

**Additional file 7: Table S3.** Characteristics at ICU admission in the training and validation sets^a^

**Additional file 8: Figure S5.** The correlation between continuous variables which were associated with the 30-day mortality of SAE patients in the multivariable logistic regression. The figure exhibited that no linear correlation was existed among the continuous variables, indicating that collinearity was not existed in the regression model.

**Additional file 9: Figure S6.** Sensitivity analysis conducted in patients in the “Martin’s criteria” cohort. In the ROC curve, the AUROC of nomogram was significantly higher than that of SOFA and LODS (A). In the calibration curve, the apparent curve and bias-corrected curve were slightly deviated from reference line, but a good conformity between observation and prediction is observed (B). In the DCA curve, medical intervention guided by the nomogram could add more net benefit than SOFA and LODS when the threshold probability (PT) between 0.1 and 0.65.

**Additional file 10: Figure S7.** Sensitivity analysis conducted in patients with GCS3-8 in the “Sepsis-3” cohort. In the ROC curve, the AUROC of nomogram was higher than that of SOFA and LODS (A). In the calibration curve, the apparent curve and bias-corrected curve were slightly deviated from reference line, but a good conformity between observation and prediction is still observed (B). In the DCA curve, medical intervention guided by the nomogram could add more net benefit than SOFA and LODS when the threshold probability (PT) between 0.1 and 0.6.

**Additional file 11: Table S4.** Comparison of models in predicting the 30-day mortality of SAE^a^ (Sensitivity analysis).

**Additional file 12: Figure S8.** The relationship between the 30-day mortality of SAE and the level of RDW. (A) The level of RDW in patients with SAE who died or survived within 30-day since ICU admission. (B) The Kaplan-Meier’s survival estimated of the 30-day survival probability of SAE patients who were divided into two groups based on the upper limit of reference interval.

## Data Availability

The datasets used and analyzed during the current study are available from the corresponding author on reasonable request.

## Abbreviations

SAE: Sepsis-associated encephalopathy
MIMIC III: Medical Information Mart for Intensive Care
GCS: Glasgow Coma Score
DCA: Decision curve analysis
BUN: Blood urea nitrogen
RDW: Red blood cell distribution width
SAPSII: The simplified acute physiology score
SOFA: Sequential Organ Failure Assessment
LODS: Logistic Organ Dysfunction System
AUROC: Area under the receiver operating characteristic curve
CNS: Central nervous system
ICD-9: International Classification of Diseases, Ninth Revision
SQL: Structure query language
CAM-ICU: Confusion Assessment Method developed for ICU patients
VIF: Variance inflation factor
IDI: Integrated discrimination improvement
CCU: Coronary care unit
CSRU: Cardiac surgical intensive care unit
MICU: Medical intensive care unit
SICU: Surgical intensive care unit
TSICU: Trauma/surgical intensive care unit
RDW: Red blood cell distribution width
PSM: Propensity score match
CRP: C-reactive protein
ESR: Erythrocyte sedimentation rate
qSOFA: Quick Sequential Organ Failure Assessment
SIRS: Systemic inflammatory response syndrome

## References

[1] Ebersoldt M, Sharshar T, Annane D (2007). Sepsis associated delirium. Intensive Care Med.

[2] Iwashyna TJ, Ely EW, Smith DM, Langa KM (2010). Long-term cognitive impairment and functional disability among survivors of severe sepsis. JAMA..

[3] Widmann CN, Heneka MT (2014). Long-term cerebral consequences of sepsis. Lancet Neurol.

[4] Eidelman LA, Putterman D, Putterman C, Sprung CL (1996). The spectrum of septic encephalopathy. Definitions, etiologies, and mortalities. JAMA.

[5] Sonneville R, de Montmollin E, Poujade J (2017). Potentially modifiable factors contributing to sepsis-associated encephalopathy. Intensive Care Med.

[6] Sprung CL, Peduzzi PN, Shatney CH (1990). Impact of encephalopathy on mortality in the sepsis syndrome. Crit Care Med.

[7] Bleck TP, Smith MC, Pierre-Louis SJC (1993). Neurologic complications of critical medical illnesses. Crit Care Med.

[8] Johnson A, Pollard T, Mark R. MIMIC-III Clinical Database. Physio Net. 2016. 10.13026/C2XW26.

[9] Johnson AEW, Pollard TJ, Shen L, et al. MIMIC-III, a freely accessible critical care database. 2016;3:160035. 10.1038/sdata.2016.35 PMC4878278 27219127

[10] Singer M, Deutschman CS, Seymour CW, et al. The third international consensus definitions for sepsis and septic shock (Sepsis-3). JAMA. 2016;315(8). 10.1001/jama.2016.0287 PMC4968574 26903338

[11] Le Gall JR, Lemeshow S, Saulnier F (1993). A New Simplified Acute Physiology Score (SAPS II) based on a European/North American multicenter study. JAMA.

[12] Ferreira FL, Bota DP, Bross A, Mélot C, Vincent JL (2001). Serial evaluation of the SOFA score to predict outcome in critically ill patients. JAMA.

[13] Martin GS, Mannino DM, Eaton S, Moss M (2003). The epidemiology of sepsis in the United States from 1979 through 2000. N Engl J Med.

[14] Sharshar T, Citerio G, Andrews PJ (2014). Neurological examination of critically ill patients: a pragmatic approach. Report of an ESICM expert panel. Intensive Care Med.

[15] Van Den Berg HA (2018). Occam’s razor: from Ockham’s via moderna to modern data science. Sci Prog.

[16] Le Gall JR, Klar J, Lemeshow S, Saulnier F, Alberti C, Artigas A, Teres D (1996). The Logistic organ dysfunction System. a new way to assess organ dysfunction in the intensive care unit. ICU Scoring Group. JAMA.

[17] Pencina MJ, D'Agostino RB, D'Agostino RB, Vasan RS (2008). Evaluating the added predictive ability of a new marker: from area under the ROC curve to reclassification and beyond. Stat Med.

[18] Collins GS, Reitsma JB, Altman DG, Moons KG (2015). Transparent reporting of a multivariable prediction model for individual prognosis or diagnosis (TRIPOD). Ann Intern Med.

[19] Ely EW, Shintani A, Truman B (2004). Delirium as a predictor of mortality in mechanically ventilated patients in the intensive care unit. JAMA..

[20] Polito A, Eischwald F, Maho ALL (2013). Pattern of brain injury in the acute setting of human septic shock. Crit Care.

[21] Peake SL, Delaney A, Bailey M (2014). Goal-directed resuscitation for patients with early septic shock. N Engl J Med.

[22] Rhodes A, Evans LE, Alhazzani W (2017). Surviving sepsis campaign: international guidelines for management of sepsis and septic shock: 2016. Intensive Care Med.

[23] Nishida O, Ogura H, Egi M (2018). The Japanese clinical practice guidelines for management of sepsis and septic shock 2016 (J-SSCG 2016). J Intensive Care.

[24] From the Centers for Disease Control (1990). Increase in national hospital discharge survey rates for septicemia--United States, 1979–1987. JAMA.

[25] Stevenson EK, Rubenstein AR, Radin GT, Wiener RS, Walkey AJ (2014). Two decades of mortality trends among patients with severe sepsis: a comparative meta-analysis. Crit Care Med.

[26] Dombrovskiy VY, Martin AA, Sunderram J, Paz HL (2005). Facing the challenge: decreasing case fatality rates in severe sepsis despite increasing hospitalizations. Crit Care Med.

[27] Brun-Buisson C, Meshaka P, Pinton P, Vallet B (2004). EPISEPSIS: a reappraisal of the epidemiology and outcome of severe sepsis in French intensive care units. Intensive Care Med.

[28] Engel C, Brunkhorst FM, Bone HG, Brunkhorst R, Gerlach H, Grond S (2007). Epidemiology of sepsis in Germany: results from a national prospective multicenter study. Intensive Care Med.

[29] Seymour CW, Liu VX, Iwashyna TJ (2016). Assessment of clinical criteria for sepsis: for the third international consensus definitions for sepsis and septic shock (Sepsis-3). JAMA..

[30] Turcato G, Serafini V, Dilda A (2016). Red blood cell distribution width independently predicts medium-term mortality and major adverse cardiac events after an acute coronary syndrome. Ann Transl Med.

[31] Sangoi MB, Da Silva SH, da Silva JE (2011). Relation between red blood cell distribution width and mortality after acute myocardial infarction. Int J Cardiol.

[32] Ani C, Ovbiagele B (2009). Elevated red blood cell distribution width predicts mortality in persons with known stroke. J Neurol Sci.

[33] Hu Z, Sun Y, Wang Q (2013). Red blood cell distribution width is a potential prognostic index for liver disease. Clin Chem Lab Med.

[34] Xu WS, Qiu XM, Ou QS (2015). Red blood cell distribution width levels correlate with liver fibrosis and inflammation: a noninvasive serum marker panel to predict the severity of fibrosis and inflammation in patients with hepatitis B. Medicine (Baltimore).

[35] Salciccioli JD, Marshall DC, Pimentel MA (2015). The association between the neutrophil-to-lymphocyte ratio and mortality in critical illness: an observational cohort study. Crit Care.

[36] Lippi G, Targher G, Montagnana M, Salvagno GL, Zoppini G, Guidi GC (2009). Relation between red blood cell distribution width and inflammatory biomarkers in a large cohort of unselected outpatients. Arch Pathol Lab Med.

[37] Hu ZD, Sun Y, Guo J (2014). Red blood cell distribution width and neutrophil/lymphocyte ratio are positively correlated with disease activity in primary Sjogren's syndrome. Clin Biochem.

[38] Hu ZD, Chen Y, Zhang L (2013). Red blood cell distribution width is a potential index to assess the disease activity of systemic lupus erythematosus. Clin Chim Acta.

[39] Vaya A, Sarnago A, Fuster O, Alis R, Romagnoli M (2015). Influence of inflammatory and lipidic parameters on red blood cell distribution width in a healthy population. Clin Hemorheol Microcirc.

[40] Perlstein TS, Weuve J, Pfeffer MA, Beckman JA (2009). Red blood cell distribution width and mortality risk in a community-based prospective cohort. Arch Intern Med.

[41] Weiss G, Goodnough LT (2005). Anemia of chronic disease. N Engl J Med.

[42] Levy MM, Fink MP, Marshall JC, Abraham E, Angus D, Cook D, Cohen J, Opal SM, Vincent JL, Ramsay G (2003). 2001 SCCM/ESICM/ACCP/ATS/SIS International Sepsis Definitions Conference. Crit Care Med.

[43] Williams JM, Greenslade JH, McKenzie JV, Chu K, Brown AFT, Lipman J (2017). Systemic inflammatory response syndrome, Quick Sequential Organ Function Assessment, and organ dysfunction: insights from a prospective database of ED patients with infection. Chest..

[44] Sedaghat S, Cremers LG, de Groot M (2016). Lower microstructural integrity of brain white matter is related to higher mortality. Neurology..

