# Supplementary figures and images for: Development of a nomogram to predict 30-day mortality of patients with sepsis-associated encephalopathy: a retrospective cohort study
Source: J Intensive Care. 2020 Jul 2;8:45. doi: 10.1186/s40560-020-00459-y (PMC7331133; doi:10.1186/s40560-020-00459-y)

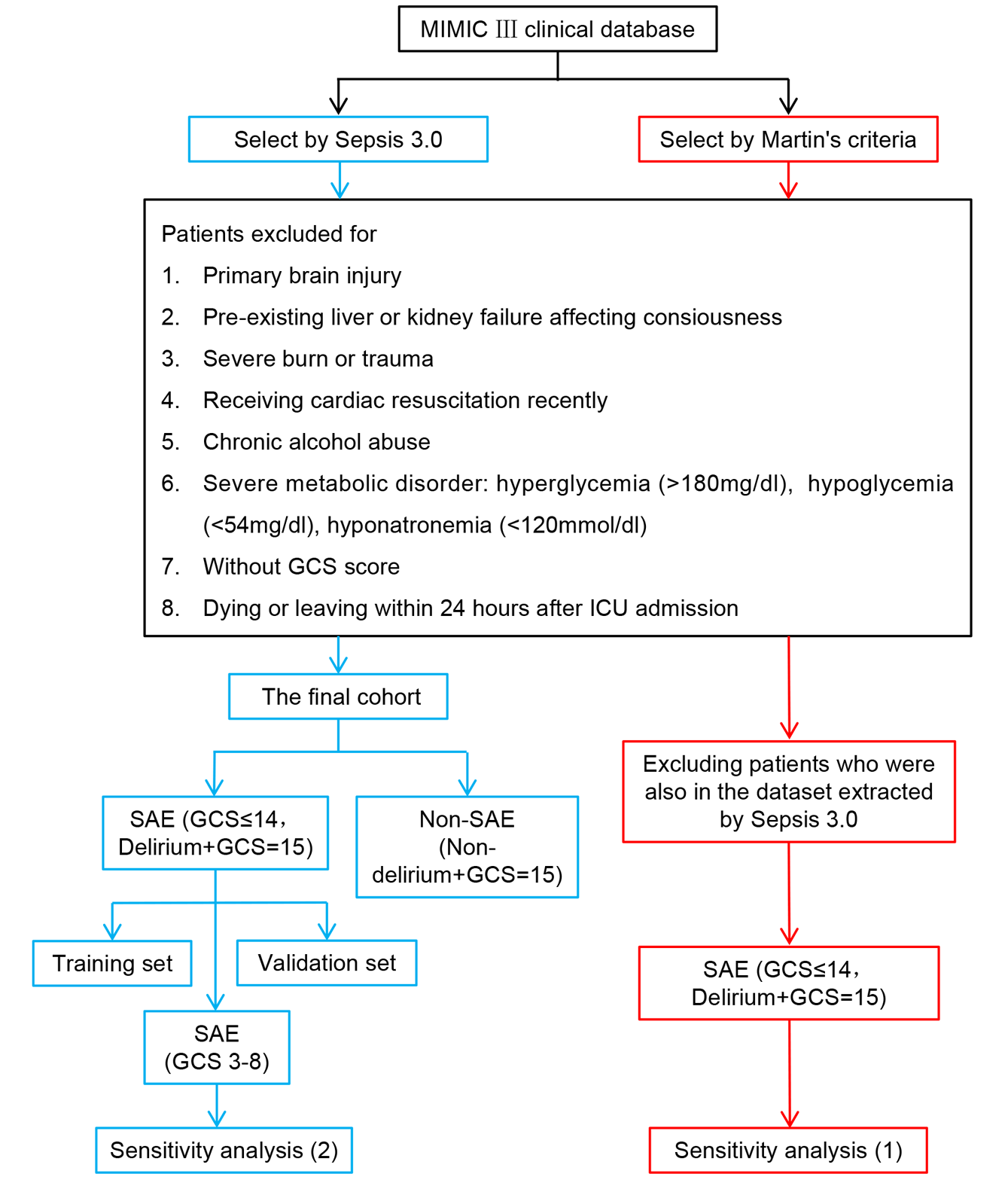

Supplement: Supplementary file 1 — Additional file 1: Figure S1. Flowchart of data extraction. Patients with sepsis were extracted from the MIMIC III database by both of Sepsis-3 and the Martin’s criteria. Then, we excluded patients with comorbidities that may have adverse impact on consciousness, or without a record of GCS, or died within 24 hours since ICU admission. The order of exclusion was consistent with what we performed by the SQL. After exclusion , patients remained in the “ Sepsis-3” cohort (blue) were picked out to make up the final cohort and those with GCS 3-8 were used to develop the nomogram and conducted sensitivity analysis, respectively. Besides, patients with SAE in the “Martin’s criteria” cohort (red) were picked out to conduct sensitivity analysis. [file 40560_2020_459_MOESM1_ESM.tif]

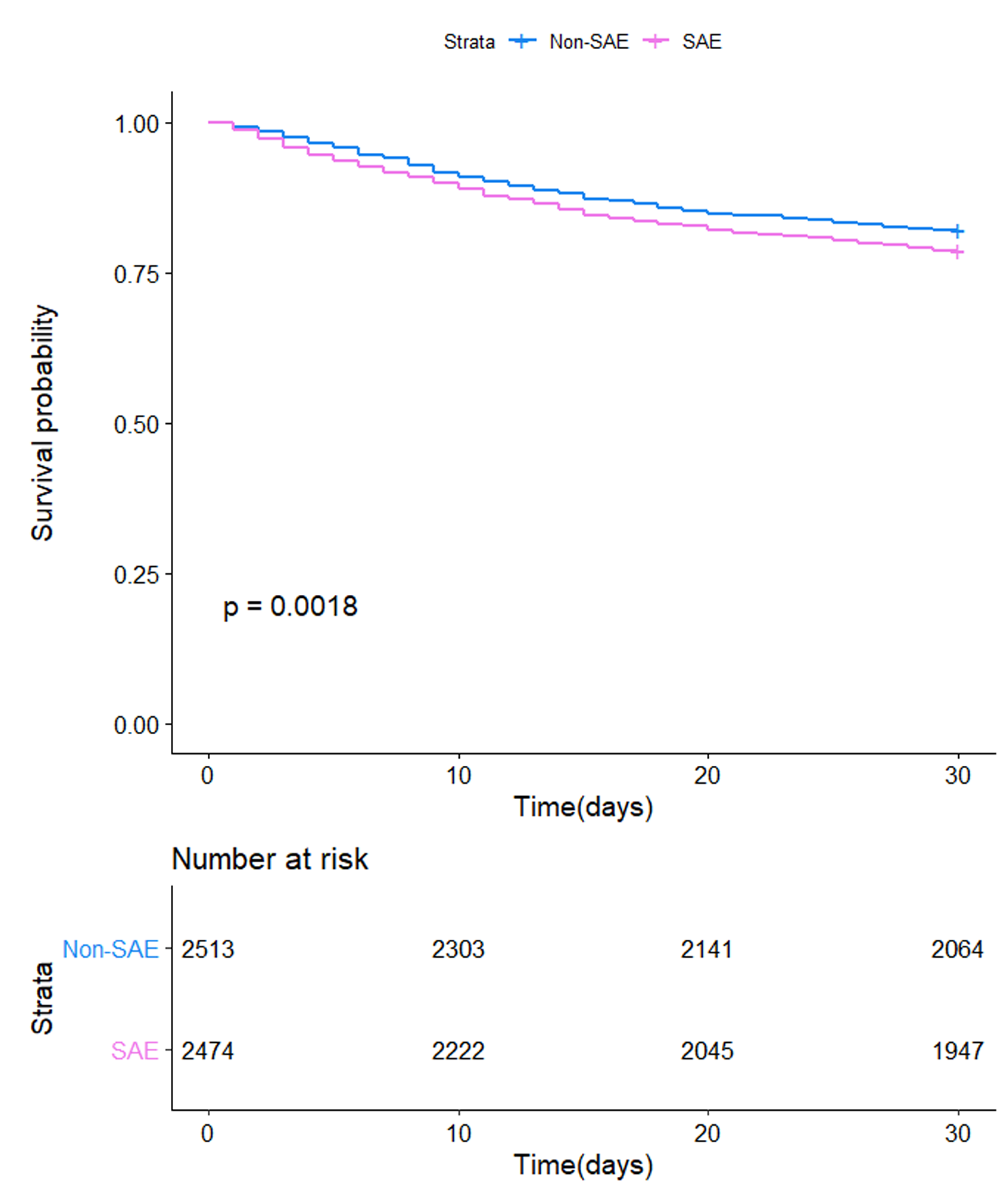

Supplement: Supplementary file 3 — Additional file 3: Figure S2. The Kaplan-Meier’s survival estimated of the 30-day survival probability of SAE and non-SAE patients. Results showed that the 30-day survival of SAE patients were significantly lower than that of non-SAE patients (Log-rank p = 0.0018). [file 40560_2020_459_MOESM3_ESM.tif]

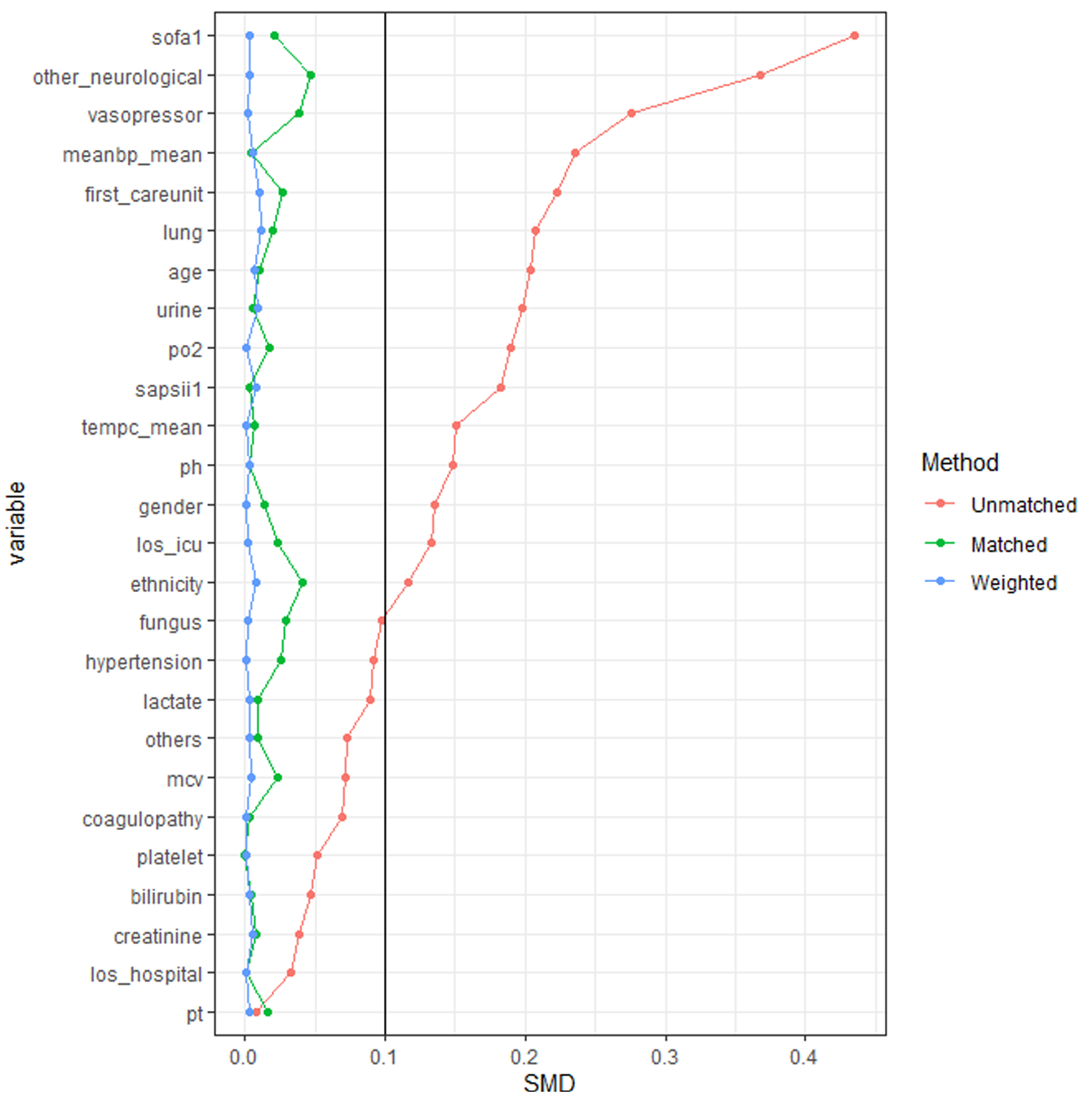

Supplement: Supplementary file 4 — Additional file 4: Figure S3. Propensity score match (PSM) between SAE and non-SAE patients. The statistically different variables in baseline characteristics and characteristics at ICU admission of patients with sepsis were exhibited as the red dot (unmatched) and all of them were matched well and similar between the two groups (green dot: matched) after PSM. [file 40560_2020_459_MOESM4_ESM.tif]

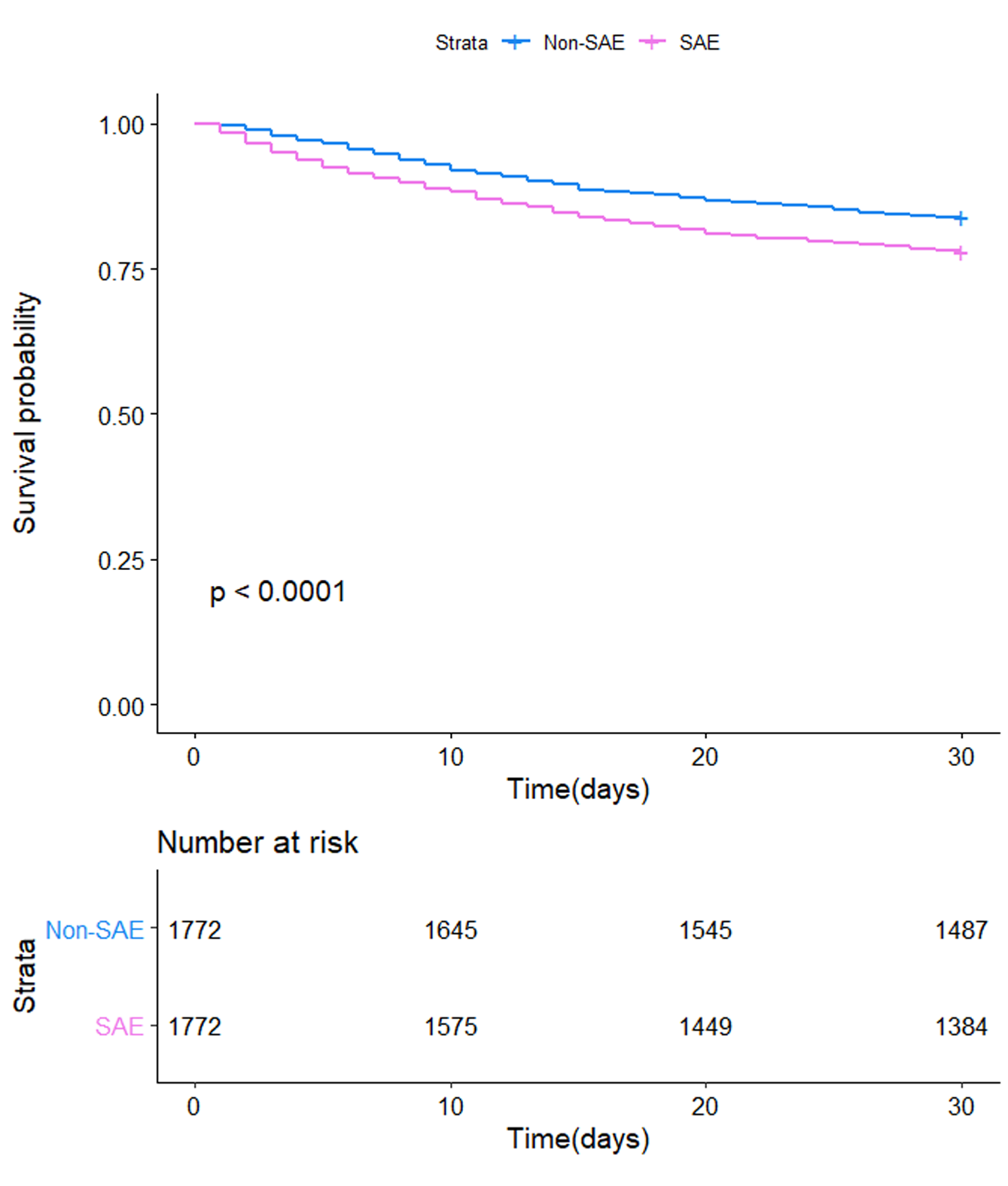

Supplement: Supplementary file 5 — Additional file 5: Figure S4. The Kaplan-Meier’s survival estimated of the 30-day survival probability of SAE and non-SAE patients after PSM. Results showed that the 30-day survival of SAE patients were significantly lower than that of non-SAE patients (Log-rank p < 0.0001). [file 40560_2020_459_MOESM5_ESM.tif]

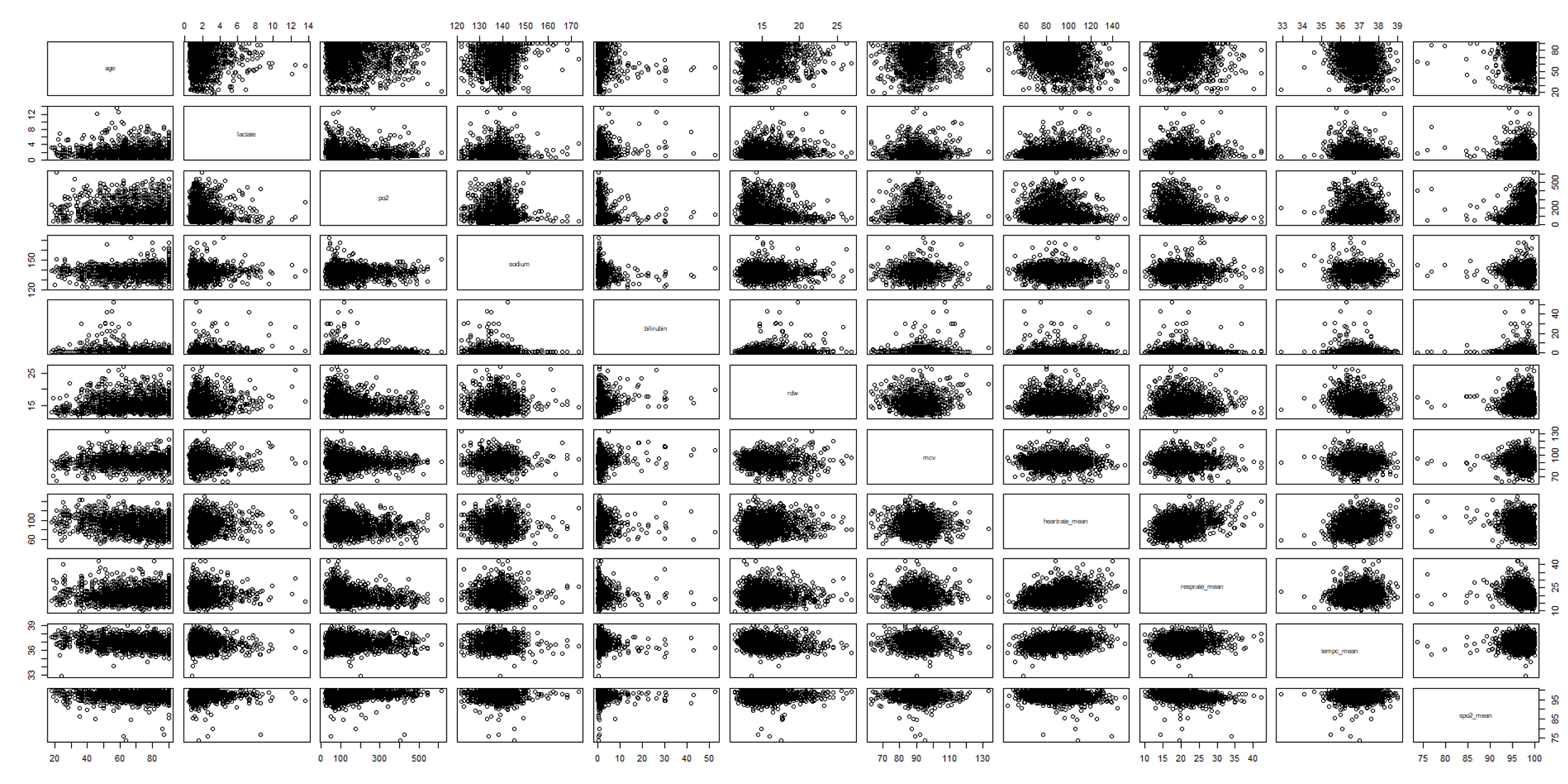

Supplement: Supplementary file 8 — Additional file 8: Figure S5. The correlation between continuous variables which were associated with the 30-day mortality of SAE patients in the multivariable logistic regression. The figure exhibited that no linear correlation was existed among the continuous variables, indicating that collinearity was not existed in the regression model. [file 40560_2020_459_MOESM8_ESM.tif]

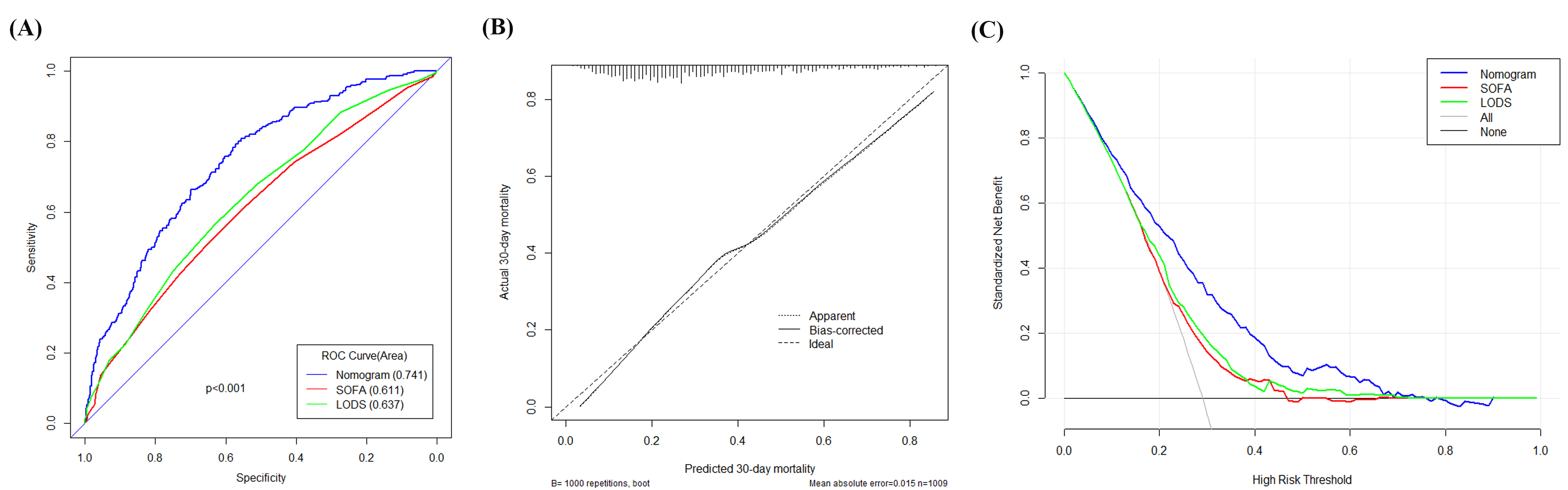

Supplement: Supplementary file 9 — Additional file 9: Figure S6. Sensitivity analysis conducted in patients in the “Martin’s criteria” cohort. In the ROC curve, the AUROC of nomogram was significantly higher than that of SOFA and LODS (A). In the calibration curve, the apparent curve and bias-corrected curve were slightly deviated from reference line, but a good conformity between observation and prediction is observed (B). In the DCA curve, medical intervention guided by the nomogram could add more net benefit than SOFA and LODS when the threshold probability (PT) between 0.1 and 0.65. [file 40560_2020_459_MOESM9_ESM.tif]

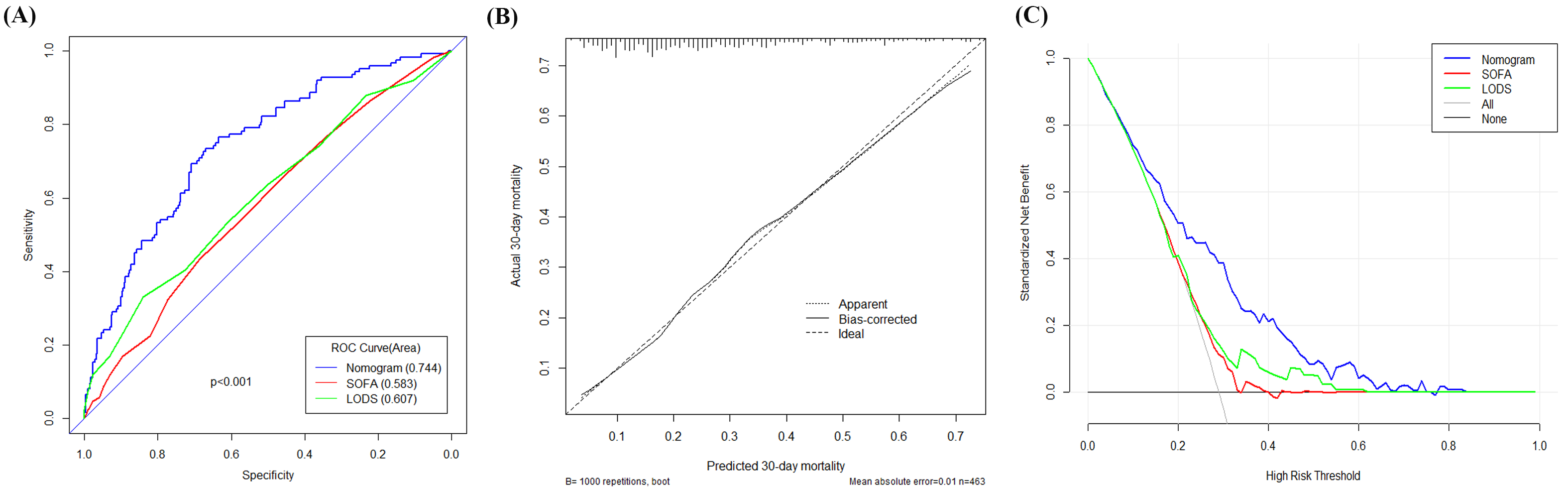

Supplement: Supplementary file 10 — Additional file 10: Figure S7. Sensitivity analysis conducted in patients with GCS3-8 in the “Sepsis-3” cohort. In the ROC curve, the AUROC of nomogram was higher than that of SOFA and LODS (A). In the calibration curve, the apparent curve and bias-corrected curve were slightly deviated from reference line, but a good conformity between observation and prediction is still observed (B). In the DCA curve, medical intervention guided by the nomogram could add more net benefit than SOFA and LODS when the threshold probability (PT) between 0.1 and 0.6. [file 40560_2020_459_MOESM10_ESM.tif]

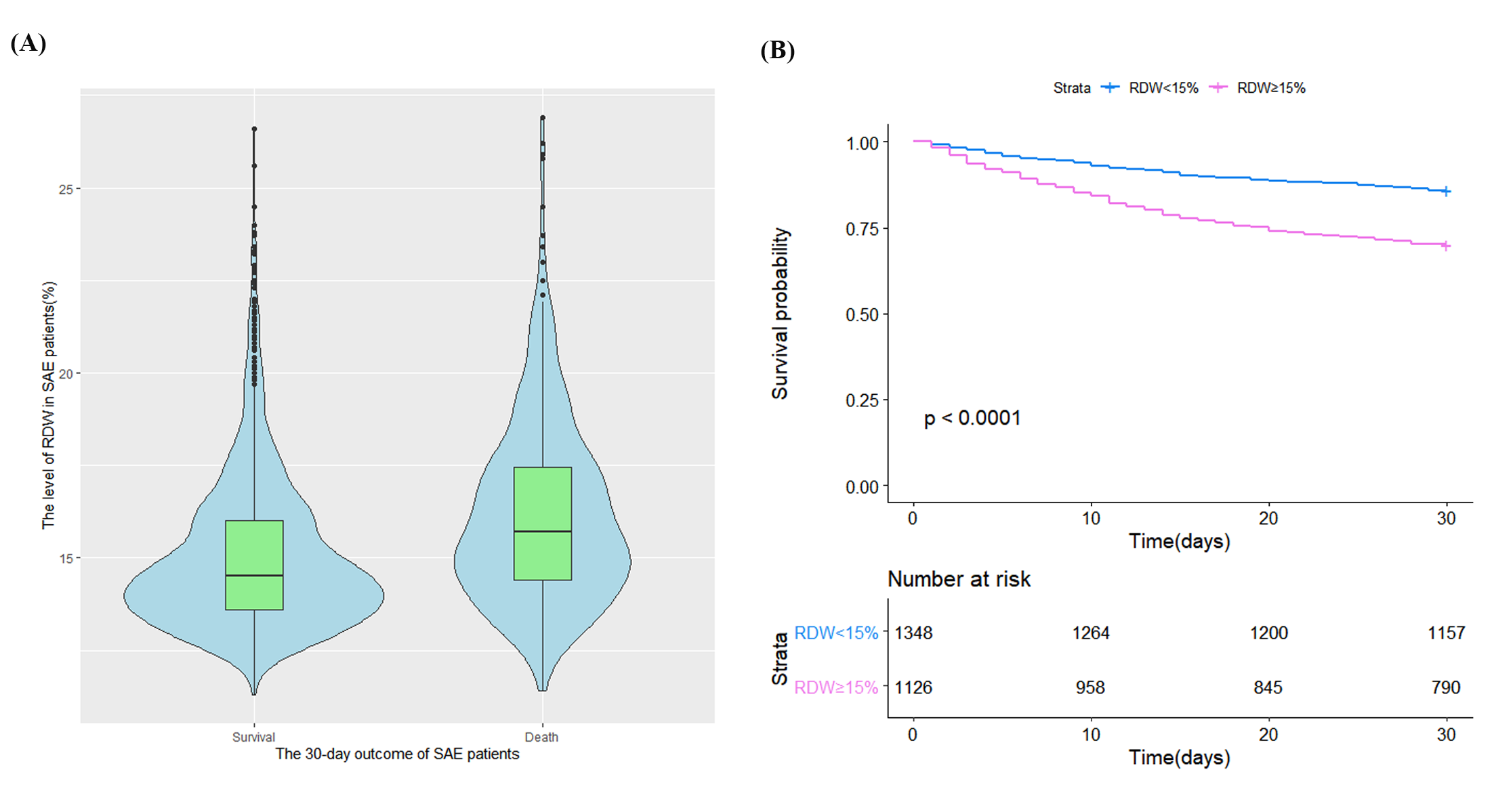

Supplement: Supplementary file 12 — Additional file 12: Figure S8. The relationship between the 30-day mortality of SAE and the level of RDW. (A) The level of RDW in patients with SAE who died or survived within 30-day since ICU admission. (B) The Kaplan-Meier’s survival estimated of the 30-day survival probability of SAE patients who were divided into two groups based on the upper limit of reference interval. [file 40560_2020_459_MOESM12_ESM.tif]
