# Supplementary material for: Development of a nomogram to predict 30-day mortality of patients with sepsis-associated encephalopathy: a retrospective cohort study
Source: J Intensive Care. 2020 Jul 2;8:45. doi: 10.1186/s40560-020-00459-y (PMC7331133; doi:10.1186/s40560-020-00459-y)
Supplement: Supplementary file 2 — Additional file 2: Table S1. Factors independently associated with 30-day mortality of patients with sepsis in the multivariate logistic analysis. [file 40560_2020_459_MOESM2_ESM.pdf]

Table S1 Factors independently associated with 30-day mortality of patients with sepsis in the multivariate logistic analysis

Variables	OR	95% CI		P value
SAE	1.26	1.07	1.49	0.005
Age (years)	1.04	1.03	1.04	<0.001
Hypertension	0.76	0.64	0.89	<0.001
Chronic pulmonary disease	1.22	1.02	1.46	0.033
Liver disease	1.39	1.02	1.87	0.034
Lactate (mmol/L)	1.13	1.07	1.19	<0.001
PO2 (mmHg)	1.00	1.00	1.00	0.006
WBC (K/uL)	1.01	1.00	1.01	0.010
Creatinine (K/uL)	0.93	0.86	0.99	0.032
BUN (K/uL)	1.01	1.00	1.01	<0.001
Hemoglobin (g/dL)	0.95	0.91	0.99	0.016
Bilirubin (EU/dL)	1.04	1.02	1.07	<0.001
RDW (%)	1.14	1.10	1.19	<0.001
MCV (fL)	1.02	1.01	1.03	<0.001
Mean heartrate (min-1)	1.01	1.00	1.01	0.006
Mean respiratory rate (min-1)	1.07	1.05	1.09	<0.001
Mean temperature (°C)	0.72	0.63	0.82	<0.001
Mean SpO2 (%)	0.96	0.92	0.99	0.014
Mechanical ventilation	1.80	1.38	2.34	<0.001
Vasopressor	2.05	1.73	2.43	<0.001

BUN, blood urea nitrogen; RDW, red blood cell distribution widths; MCV, mean corpuscular volume.

Hosmer-Lemeshow test: p=0.853.
