# Supplementary material for: Development of a nomogram to predict 30-day mortality of patients with sepsis-associated encephalopathy: a retrospective cohort study
Source: J Intensive Care. 2020 Jul 2;8:45. doi: 10.1186/s40560-020-00459-y (PMC7331133; doi:10.1186/s40560-020-00459-y)
Supplement: Supplementary file 6 — Additional file 6: Table S2. Baseline characteristics of patients in the training and validation setsa. [file 40560_2020_459_MOESM6_ESM.pdf]

Table S2 Baseline characteristics of patients in the training and validation sets [a]

Variable	SAE patients<br>n=2474	Training set<br>n=1731	Validation set<br>n=743	P value
Age, years	73 [58, 83]	73 [57, 83]	73 [58.5, 83.0]	0.654
Gender, male	1246 (50.36)	864 (49.91)	382 (51.41)	0.522
**Ethnicity, n(%)**				0.873
White	1855 (74.98)	1291 (74.58)	564 (75.91)	
Black	214 (8.65)	148 (8.55)	66 (8.88)	
Hispanic or Latino	67 (2.71)	49 (2.83)	18 (2.42)	
Asian	50 (2.02)	37 (2.14)	13 (1.75)	
Others	288 (11.64)	206 (11.90)	82 (11.04)	
**Comorbidity, n(%)**				
Cardiovascular diseases	1359 (54.93)	943 (54.48)	416 (55.99)	0.517
Peripheral vascular disease	253 (10.23)	180 (10.40)	73 (9.83)	0.476
Other neurological Diseases	494 (19.97)	350 (20.22)	144 (19.39)	0.672
Hypertension	1276 (51.58)	886 (51.18)	390 (52.49)	0.581
Chronic Pulmonary Disease	543 (21.95)	375 (21.66)	168 (22.61)	0.639
Diabetes	460 (18.59)	308 (17.79)	152 (20.46)	0.132
Hypothyroidism	307 (12.41)	210 (12.13)	97 (13.06)	0.567
Liver Disease	206 (8.33)	149 (8.61)	57 (7.67)	0.488
Coagulopathy	404 (16.33)	283 (16.35)	121 (16.29)	1.00
Anemias	678 (27.41)	478 (27.61)	200 (26.92)	0.759

[a] Continuous data are presented as median (interquartile range), whereas categorical data are presented as frequency (percentage).
