# Supplementary material for: Development of a nomogram to predict 30-day mortality of patients with sepsis-associated encephalopathy: a retrospective cohort study
Source: J Intensive Care. 2020 Jul 2;8:45. doi: 10.1186/s40560-020-00459-y (PMC7331133; doi:10.1186/s40560-020-00459-y)
Supplement: Supplementary file 7 — Additional file 7: Table S3. Characteristics at ICU admission in the training and validation setsa [file 40560_2020_459_MOESM7_ESM.pdf]

Table S3 Characteristics at ICU admission in the training and validation sets [a]

Variable	SAE patients n=2474	Training set n=1731	Validation set n=743	P value
Hospital stay time, days	10.9 [6.4, 19.6]	11.2 [6.4, 19.6]	10.7 [6.5, 19.7]	0.828
ICU stay time, days	3 [1.8, 6.3]	3.1 [1.8, 6.6]	3.0 [1.7, 6.0]	0.719
30-day mortality, n (%)	527 (21.30)	368 (21.26)	159 (21.40)	0.980
Mechanical ventilation, n(%)	209 (8.45)	153 (8.84)	56 (7.54)	0.323
Vasopresser, n (%)	577 (23.33)	421 (24.32)	156 (21.00)	0.082
**First careunit，n(%)**				0.489
CCU	233 (9.42)	164 (9.47)	69 (9.29)	
CSRU	233 (9.01)	147 (8.49)	76 (10.23)	
MICU	1295 (52.34)	918 (53.03)	377 (50.74)	
SICU	413 (16.69)	293 (16.93)	120 (16.15)	
TSICU	310 (12.53)	209 (12.07)	101 (13.59)	
**Severe Score [b]**				
Modified SOFA	3 [2,5]	3 [2,6]	3 [2,5]	0.415
Modified SAPSII	37.44±11.97	37.42±11.81	37.48±12.39	0.916
GCS score	11.45±3.21	11.42±3.22	11.52±3.19	0.464
**Vital signs [c]**				
Mean heartrate (min^{-1})	89.25±16.54	89.33±16.54	89.08±16.56	0.726
Mean arterial pressure (mmHg)	75.63±10.91	75.57±10.60	75.76±11.59	0.709
Mean respiratory rate (min^{-1})	20.07±4.31	20.10±4.37	20.00±4.15	0.586
Mean temperature(°C)	36.87±0.66	36.88±0.66	36.87±0.66	0.860
Mean SpO2(%)	97.3 [96.0, 98.4]	97.2 [95.9, 98.4]	97.4 [96.0, 98.5]	0.212
**Laboratory tests [d]**				
Lactate (mmol/L)	1.5 [1.1, 2.2]	1.5 [1.1, 2.3]	1.6 [1.1, 2.2]	0.459
PCO_2 (mmHg)	40 [35,47]	40 [35,47]	40 [35,47]	0.804
PO_2 (mmHg)	103 [73,175]	104 [73,179]	100 [73,166]	0.449
PH	7.378±0.092	7.377±0.092	7.380±0.092	0.514
Creatinine (K/uL)	1.1 [0.8, 1.6]	1.1 [0.8, 1.6]	1.1 [0.8, 1.7]	0.236
BUN (K/uL)	23 [15,38]	23 [15,38]	23 [15,37]	0.373
ALT [e]	1.4 [1.2, 1.7]	1.4 [1.2, 1.7]	1.4 [1.2, 1.7]	0.726
AST [f]	1.5 [1.3, 1.8]	1.5 [1.3, 1.8]	1.5 [1.3, 1.8]	0.708
Bilirubin (EU/dL)	0.6 [0.4, 1.1]	0.6 [0.4, 1.1]	0.6 [0.4, 1.1]	0.853
Hemoglobin (g/dL)	11.49±2.19	11.50±2.18	11.47±2.21	0.758
Platelet (K/uL)	232 [167,308]	231 [164.5, 307.0]	238 [170.0, 310.5]	0.330
Potassium (K/uL)	4.2 [3.8, 4.7]	4.2 [3.8, 4.7]	4.2 [3.8, 4.6]	0.242
Sodium (K/uL)	138 [135,141]	138 [135,141]	138 [135,141]	0.937
PT (sec)	13.8 [12.8, 16.0]	13.9 [12.8, 16.1]	13.7 [12.8, 15.9]	0.270
RDW (%)	15.29±2.21	15.30±2.23	15.26±2.17	0.621
WBC (K/uL)	10.8 [7.3, 15.1]	10.7 [7.30, 15.05]	10.8 [7.3, 15.3]	0.803
Lymphocyte (%)	9.6 [5.8, 16.3]	9.8 [5.9, 16.5]	9.0 [5.55, 16.10]	0.238

Neutrophil (%)	81.15 [72.13, 88.00]	81.2 [72, 88]	81 [72.85, 88.75]	0.580
MCV (fL)	91 [86,95]	90 [86,95]	91 [86,95]	0.151
**Infection site, n(%)**				
Urine	1224 (49.47)	847 (48.93)	377 (50.74)	0.435
Blood	640 (25.87)	438 (25.30)	202 (27.19)	0.352
Lung	825 (33.35)	579 (33.45)	246 (33.11)	0.906
Catheter	114 (4.61)	85 (4.91)	29 (3.90)	0.322
Gastrointestinal tract	154 (6.22)	97 (5.60)	57 (7.67)	0.063
Abdominal cavity	55 (2.22)	37 (2.14)	18 (2.42)	0.770
Skin/Soft tissue	424 (17.14)	289 (16.70)	135 (18.17)	0.405
Others	68 (2.75)	44 (2.54)	24 (3.23)	0.409
**Microorganisms, n(%)**				
Gram-positive	1265 (51.13)	888 (51.30)	377 (50.74)	0.833
Gram-negative	984 (39.77)	678 (39.17)	306 (41.18)	0.371
Fungus	699 (28.25)	487 (28.13)	212 (28.53)	0.878

[a] Parametric continuous data are presented as mean ± standard deviation (SD), non-parametric continuous data are presented as median (interquartile ranges), whereas categorical data are presented as frequency (percentage)

[b] Severe score is calculated on the first day of each ICU patients' stay

[c] Vital signs is calculated on the first 24 hours of each ICU patients' stay

[d] Laboratory tests recorded the first result of each patients' ICU stay

[e] ALT in the table is the value after logarithmic transformation

[f] ALT in the table is the value after logarithmic transformation

CCU, coronary care unit; CSRU, cardiac surgical intensive care unit; MICU, medical intensive care unit; SICU, surgical intensive care unit; TSICU, trauma/surgical intensive care unit; SOFA, sequential organ failure assessment; SAPSII, the simplified acute physiology socre; GCS, the Glasgow Coma Score; RDW, red blood cell distribution widths; MCV, mean corpuscular volume.
