# Supplementary material for: Development of a nomogram to predict 30-day mortality of patients with sepsis-associated encephalopathy: a retrospective cohort study
Source: J Intensive Care. 2020 Jul 2;8:45. doi: 10.1186/s40560-020-00459-y (PMC7331133; doi:10.1186/s40560-020-00459-y)
Supplement: Supplementary file 11 — Additional file 11: Table S4. Comparison of models in predicting the 30-day mortality of SAEa (Sensitivity analysis). [file 40560_2020_459_MOESM11_ESM.pdf]

Table S4 Comparison of models in predicting the 30-day mortality of SAE[a] (Sensitivity analysis)

Predictive Model		AUROC	P value	IDI	P value	Brier index	P value
**Sensitivity(1)**	Nomogram	0.741 [0.709-0.773]				0.182[0.170-0.195]	
	SOFA	0.611 [0.573-0.648]	<0.001	0.122 [0.101-0.144]	<0.001	0.208 [0.197-0.219]	<0.001
	LODS	0.637 [0.601-0.674]	<0.001	0.103 [0.079-0.127]	<0.001	0.204 [0.193-0.216]	<0.001
**Sensitivity(2)**	Nomogram	0.744 [0.694-0.794]				0.167 [0.148-0.186]	
	SOFA	0.583 [0.525-0.640]	<0.001	0.135 [0.103-0.166]	<0.001	0.193 [0.174-0.212]	<0.001
	LODS	0.607 [0.548-0.667]	<0.001	0.113 [0.082-0.144]	<0.001	0.188 [0.169-0.207]	<0.001

[a]The P value was drew by comparing the results of nomogram with SOFA or LODS.

SOFA, sequential organ failure assessment; AUROC, area under the receiver operating characteristic curve; IDI, integrated discrimination improvement.
